# Once‐Nightly Sodium Oxybate Meets American Academy of Sleep Medicine Criteria for Treatment of Narcolepsy

**DOI:** 10.1111/jsr.70189

**Published:** 2025-08-25

**Authors:** Luis E. Ortiz, Anne Marie Morse, Michael J. Thorpy, Clete A. Kushida, John Harsh, Thomas Roth, Jennifer Gudeman, Yves Dauvilliers

**Affiliations:** ^1^ Johns Hopkins Medical Institutions, Johns Hopkins All Children's Hospital St. Petersburg Florida USA; ^2^ Geisinger Commonwealth School of Medicine, Geisinger Medical Center, Janet Weis Children's Hospital Danville Pennsylvania USA; ^3^ Albert Einstein College of Medicine, Montefiore Medical Center New York New York USA; ^4^ Stanford University School of Medicine Stanford California USA; ^5^ Colorado Sleep Institute Boulder Colorado USA; ^6^ Sleep Disorders and Research Center, Henry Ford Health System Detroit Michigan USA; ^7^ Avadel Pharmaceuticals Chesterfield Missouri USA; ^8^ Sleep‐Wake Disorders Center, Department of Neurology, Gui‐De‐Chauliac Hospital, Institute for Neurosciences of Montpellier INM, INSERM University of Montpellier Montpellier France

**Keywords:** clinical improvement cutoff, excessive daytime sleepiness, FT218, LUMRYZ, MCID, minimal clinically important difference

## Abstract

Data from the REST‐ON trial were not available before the 2021 American Academy of Sleep Medicine (AASM) clinical practice guideline update, which included a literature review through August 2020. This post hoc analysis from REST‐ON assessed participants who achieved clinically significant improvements on individual AASM clinical significance thresholds (CSTs). Composites of the coprimary endpoints and a secondary endpoint were also analysed. Participants with narcolepsy aged ≥ 16 years were randomised 1:1 to once‐nightly sodium oxybate (ON‐SXB) or placebo for 13 weeks. Coprimary endpoints were mean sleep latency on the Maintenance of Wakefulness Test (MWT), Clinical Global Impression of Improvement (CGI‐I) rating, and number of cataplexy episodes; secondary endpoints included the Epworth Sleepiness Scale (ESS) score. Outcomes with ON‐SXB treatment compared with baseline were assessed according to the CSTs, and for those who met CSTs, the proportions of participants who experienced clinically significant improvements on a composite of ≥ 2, ≥ 3, or 4 endpoints were calculated. For improvements from baseline with ON‐SXB at Week 13 (9‐g dose), mean sleep latency on the MWT increased 10.8 min (CST, ≥ 2‐min increase), 92.8% rated improvement on the CGI‐I (CST, ≥ 33% of participants reporting improvement), reduction in number of cataplexy episodes was 60.8% reduction (CST, ≥ 25% reduction), and reduction in ESS score was −6.5 (CST, ≥ 2‐point decrease). At Weeks 3, 8 and 13, significantly more participants treated with ON‐SXB versus placebo experienced clinical improvements on ≥ 2, ≥ 3, or 4 endpoints (*p* ≤ 0.05). These data demonstrate the robust efficacy of ON‐SXB across multiple clinically important narcolepsy symptoms per established CSTs, further supporting the use of ON‐SXB in clinical practice.

**Trial Registration:** This manuscript presents the results of a post hoc analysis from the REST‐ON clinical trial (NCT02720744).

## 1 Introduction

Narcolepsy is a rare, chronic neurologic disorder characterised by excessive daytime sleepiness (EDS) that can occur with cataplexy (narcolepsy Type 1 [NT1]) or without cataplexy (narcolepsy Type 2 [NT2]) (Acquavella et al. 2020; Kornum et al. 2017). Additional hallmark symptoms of narcolepsy include disrupted nighttime sleep (DNS), sleep paralysis, and hypnagogic/hypnopompic hallucinations (Dauvilliers et al. 2007; Kornum et al. 2017). The American Academy of Sleep Medicine (AASM) clinical practice guidelines (CPGs) have recommended sodium oxybate (SXB), the sodium salt of γ‐hydroxybutyrate, for treatment of narcolepsy symptoms since 2007 (Jazz Pharmaceuticals 2023a; Morgenthaler et al. 2007). As part of the 2021 update to the AASM guidelines, the AASM task force conducted a systematic review and meta‐analysis and developed clinical significance thresholds (CSTs) of critical narcolepsy outcome tools to determine the strength of recommendation of narcolepsy treatment options (Maski et al. 2021b). The CPGs strongly recommend SXB for the treatment of EDS and cataplexy in adults with narcolepsy (Maski et al. 2021a). The guidelines developed by the European Narcolepsy Network, European Academy of Neurology, and European Sleep Research Society also strongly recommend the use of SXB for EDS, cataplexy, and DNS (Bassetti et al. 2021).

Extended‐release once‐nightly SXB (ON‐SXB; LUMRYZ, sodium oxybate for extended‐release oral suspension [FT218]) was approved by the US Food and Drug Administration (FDA) for the treatment of cataplexy or EDS in adults in 2023 and in paediatric patients aged ≥ 7 years with narcolepsy in 2024, after the AASM guidelines were released (Avadel CNS Pharmaceuticals 2024). This formulation of SXB is taken once at bedtime, eliminating the middle‐of‐the‐night dose required with twice‐nightly oxybates (Avadel CNS Pharmaceuticals 2024; Jazz Pharmaceuticals 2023a; Jazz Pharmaceuticals 2023b). ON‐SXB demonstrated efficacy and safety in the pivotal phase 3 REST‐ON clinical trial (Kushida et al. 2022). Treatment with ON‐SXB at Weeks 3 (6 g), 8 (7.5 g) and 13 (9 g) led to statistically significant improvement versus placebo (*p* < 0.001) on the coprimary endpoints of mean sleep latency on the Maintenance of Wakefulness Test (MWT), Clinical Global Impression of Improvement (CGI‐I) rating, and number of weekly cataplexy episodes and the secondary endpoint of the Epworth Sleepiness Scale (ESS) score. ON‐SXB was well tolerated by participants in REST‐ON; the most common adverse events aligned with the reported safety profile of twice‐nightly SXB (Jazz Pharmaceuticals 2023a) and included dizziness, nausea, vomiting, headache and enuresis (Kushida et al. 2022).

Data from the REST‐ON trial were published after the cutoff date for inclusion into the 2021 AASM CPG update (Kushida et al. 2022; Maski et al. 2021b). The AASM task force identified > 30 CSTs for critical and important measures and was transparent in the application of these CSTs for their evidence‐based recommendations. The CSTs available for outcome measures from REST‐ON were ≥ 2 min increase in mean sleep latency on the MWT, ≥ 33% of patients reporting improvement or ≥ 1‐point decrease in CGI, ≥ 25% reduction in the number of weekly cataplexy episodes, and ≥ 2‐point decrease in ESS score. These thresholds apply to both the difference in treatment effect versus placebo and the change from baseline with treatment. AASM defined these CSTs as the minimum improvement considered clinically meaningful to clinicians and patients. Here, we analyse the previously published results from the REST‐ON trial (Kushida et al. 2022) according to the 2021 AASM CSTs. Separate from the AASM CST analysis, we also present a composite of the coprimary endpoints and a secondary endpoint measure of EDS, recognising that clinicians manage complex symptomatology in narcolepsy as opposed to a single endpoint.

## 2 Methods

### 2.1 Study Design and Participants

REST‐ON was a randomised, double‐blind, placebo‐controlled, multicenter Phase 3 clinical trial that assessed the efficacy and safety of ON‐SXB in participants with narcolepsy; detailed methodology has been previously published (Kushida et al. 2022). Individuals aged ≥ 16 years with documented NT1 or NT2 and EDS for > 3 months, an ESS score > 10, and cataplexy for > 3 months (NT1 only) were eligible. For randomisation, participants were required to have a mean sleep latency of < 11 min on the MWT and current continuing presence of cataplexy, defined as an average of eight cataplexy episodes per week (NT1 only). Participants were randomised 1:1 to receive ON‐SXB or placebo according to the dosing schedule of 4.5 g for 1 week, 6 g for 2 weeks, 7.5 g for 5 weeks, and 9 g for 5 weeks. Concomitant use of alerting agents was permitted if the dose was stable for ≥ 3 weeks prior to screening. Coprimary endpoints were change from baseline in mean sleep latency on the MWT, CGI‐I rating, and number of cataplexy episodes. Change from baseline in ESS score was a secondary endpoint.

Institutional review boards approved the protocol of the REST‐ON trial. The trial was conducted in compliance with the ethical principles of the Declaration of Helsinki, Good Clinical Practice guidelines, International Council for Harmonisation guidelines, applicable national and local laws, and regulatory requirements. All adult participants (aged ≥ 18 years) provided written informed consent. If participants were aged 16 or 17 years, both the participant and their legally authorised representative provided informed consent.

### 2.2 Assessments

#### 2.2.1 MWT

The MWT was used to measure participants' mean sleep latency in minutes across five trials of up to 30 min each.

#### 2.2.2 CGI‐I

The CGI‐I is a clinician‐rated assessment that was used to evaluate the global improvement of a participant, rated on a 7‐point scale from 1 (very much improved) to 7 (very much worse). At baseline, participants completed the Clinical Global Impression of Sleepiness, which assessed the severity of their sleepiness on a 7‐point scale from 1 (normal, not at all sleepy) to 7 (among the most extremely sleepy).

#### 2.2.3 Number of Weekly Cataplexy Episodes

In a daily sleep and symptom diary, participants with NT1 reported the number of cataplexy episodes that occurred each day. A minimum of three entries per week was required for the week to be considered valid. The mean weekly number of cataplexy episodes was calculated as the number of cataplexy episodes divided by the number of days with available data of valid weeks within that period, then multiplied by seven.

#### 2.2.4 ESS

The ESS is a validated questionnaire that was used to evaluate the extent of the participant's sleepiness in everyday situations (Johns 1991). Participants rated their likelihood of dozing or falling asleep during 8 activities on a scale from 0 (never) to 3 (high).

### 2.3 Statistical Analysis

Data from the modified intent‐to‐treat (mITT) population, which included all randomised participants who underwent ≥ 1 efficacy measurement after receiving the 6‐g dose of ON‐SXB, were analysed across all endpoints. Least squares mean (LSM) differences with ON‐SXB versus placebo were calculated for change from baseline in mean sleep latency on the MWT, percentage reduction in the number of cataplexy episodes for participants with NT1, and ESS score at each time point using a mixed‐effects model for repeated measures. Fixed effects included in the model were treatment, visit at which the measurement was taken, treatment‐by‐visit interaction, site (US vs. non‐US), and baseline score as a covariate; participants were included in the model as random effects.

The percentage of participants with improvement, defined as a rating of very much, much, or minimally improved on the CGI‐I, was calculated for each dose. A Generalised Linear Mixed Model (GLIMMIX) with a logit link function was used to analyse the proportion of participants with improvement on the CGI‐I. The observed values for the categorised response were used as responses in the model. The model used treatment, visit at which the measurement was taken, treatment‐by‐visit interaction, and site (US vs. non‐US) as fixed effects and participant as a random effect. AASM CSTs included a ≥ 2‐min increase on the MWT, ≥ 33% reporting improvement on the CGI‐I, ≥ 25% decrease in weekly cataplexy episodes, and ≥ 2‐point decrease in ESS score. The percentage of participants who met the CST in the mITT population and those with NT1 or NT2 were calculated for each outcome. The proportion of participants with NT1 who experienced clinically significant improvements on a composite of ≥ 2, ≥ 3, or 4 endpoints was calculated. Two‐sided *p* values were calculated using a Fisher's exact test for the proportion of participants who had clinically significant improvements on individual and composite endpoints. Although data are presented as percentages to give readers a frame of reference for the results, the analyses were performed on absolute numbers.

## 3 Results

### 3.1 Participants

Of the 222 randomised participants in REST‐ON, 212 received ≥ 1 dose of ON‐SXB (*n* = 107) or placebo (*n* = 105). Baseline demographics were similar between the treatment arms (Table 1), and 76.4% of participants had NT1. The following results are presented from the mITT population, which included 190 participants (ON‐SXB, *n* = 97; placebo, *n* = 93).

**TABLE 1:** Baseline demographics and disease characteristics.

Characteristic	ON‐SXB *n* = 107	Placebo *n* = 105
Mean age (range), years	30.9 (16–72)	31.6 (16–69)
Sex, *n* (%)		
Female	69 (64.5)	75 (71.4)
Male	38 (35.5)	30 (28.6)
Race, *n* (%)		
White	80 (74.8)	80 (76.2)
Black/African American	21 (19.6)	15 (14.3)
Asian	3 (2.8)	8 (7.6)
Other^a^	3 (2.8)	2 (1.9)
Region, *n* (%)		
United States	63 (58.9)	53 (50.5)
Rest of world	44 (41.1)	52 (49.5)
Median BMI (range), kg/m^2^	26.1 (16.9–71.9)	26.4 (18.1–46.5)
Narcolepsy type, *n* (%)		
NT1	80 (74.8)	82 (78.1)
NT2	27 (25.2)	23 (21.9)
Concomitant alerting agent use, *n* (%)	72 (67.3)	58 (55.2)

*Note*: Table adapted from Kushida et al. 2022. *Sleep* 45 (6):zsab200.

Abbreviations: BMI, body mass index; NT1, narcolepsy type 1; NT2, narcolepsy type 2; ON‐SXB, once‐nightly sodium oxybate.

^a^ Egyptian (*n* = 2); white, American Indian/Alaska Native (*n* = 1); half Asian, half white (*n* = 1); and multiracial (white/African American/Native American, *n* = 1).

### 3.2 Efficacy

#### 3.2.1 MWT

**FIGURE 1:**
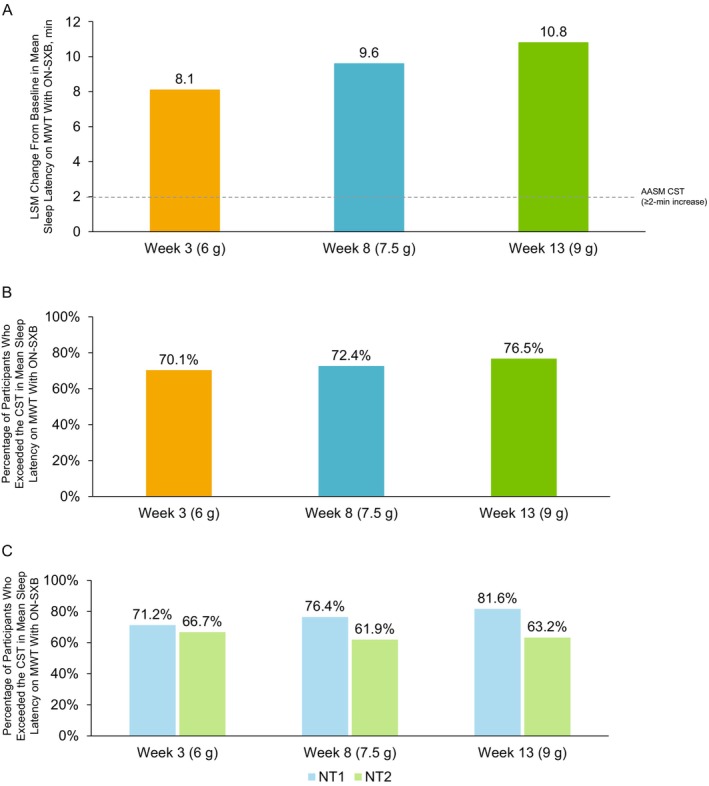
Clinically significant improvements in mean sleep latency on the MWT with ON‐SXB. LSM change from baseline with ON‐SXB in mean sleep latency on MWT with the associated CST (A), percentage of participants who exceeded the CST in the mITT population (B), and percentage of participants who exceeded the CST in the mITT population by narcolepsy type (C). AASM CST is shown as a dotted line. AASM, American Academy of Sleep Medicine; CST, clinical significance threshold; LSM, least squares mean; mITT, modified intent‐to‐treat; MWT, Maintenance of Wakefulness Test; NT1, narcolepsy type 1; NT2, narcolepsy type 2; ON‐SXB, once‐nightly sodium oxybate.

The LSM change (standard error [SE]) from baseline in mean sleep latency on the MWT with ON‐SXB at Week 3 (6 g), Week 8 (7.5 g) and Week 13 (9 g) was 8.1 (0.75), 9.6 (0.86) and 10.8 (0.96) minutes, respectively, exceeding the AASM CST (Figure 1A). Statistically significant improvements were observed with ON‐SXB versus placebo (*p* < 0.001); LSM difference (95% confidence interval [CI]) in change from baseline at Week 3 (6 g), Week 8 (7.5 g), and Week 13 (9 g) was 5.0 (2.9–7.1), 6.2 (3.8–8.6) and 6.1 (3.5–8.8) minutes, respectively, with ON‐SXB versus placebo, which also exceeded the AASM CST criteria. The percentage of participants who received ON‐SXB and exceeded the CST was 70.1% (61/87) at Week 3 (6 g), 72.4% (55/76) at Week 8 (7.5 g) and 76.5% (52/68) at Week 13 (9 g) (Figure 1B). The proportion of participants with NT1 who exceeded the CST was 71.2% (47/66) at Week 3 (6 g), 76.4% (42/55) at Week 8 (7.5 g) and 81.6% (40/49) at Week 13 (9 g; Figure 1C). The proportion of participants with NT2 who exceeded the CST was 66.7% (14/21) at Week 3 (6 g), 61.9% (13/21) at Week 8 (7.5 g) and 63.2% (12/19) at Week 13 (9 g). Results for mean sleep latency on the MWT with the placebo group are presented in the (Figure S1A–C).

#### 3.2.2 CGI‐I

**FIGURE 2:**
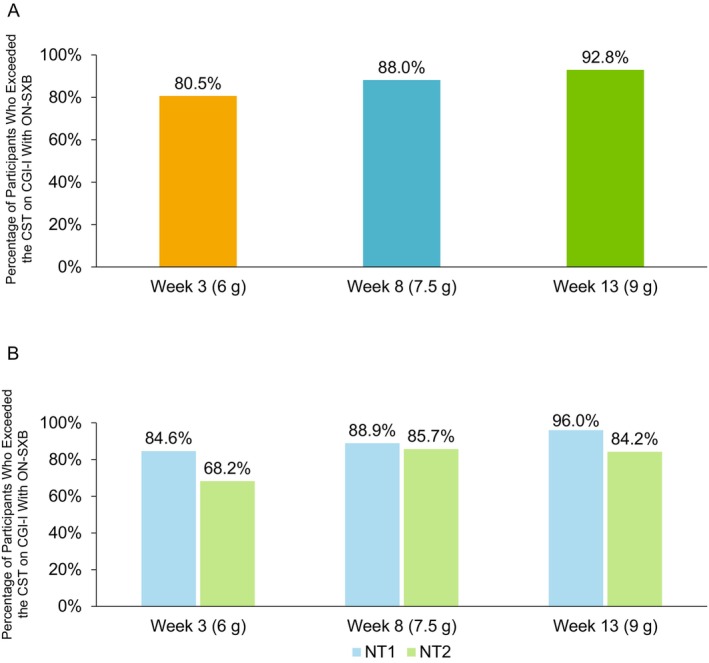
Clinically significant improvements in CGI‐I rating with ON‐SXB. Percentage of participants with very much, much, or minimal improvement on CGI‐I with ON‐SXB and the associated CST in the mITT population (A) and percentage of participants who exceeded the CST in the mITT population by narcolepsy type (B). AASM, American Academy of Sleep Medicine; CGI‐I, Clinical Global Impression of Improvement; CST, clinical significance threshold; mITT, modified intent‐to‐treat; NT1, narcolepsy type 1; NT2, narcolepsy type 2; ON‐SXB, once‐nightly sodium oxybate.

The percentage of participants with improvement on the CGI‐I with ON‐SXB was 80.5% (70/87) at Week 3 for the 6‐g dose, 88.0% (66/75) at Week 8 for the 7.5‐g dose and 92.8% (64/69) at Week 13 for the 9‐g dose (Figure 2A). Thus, all tested ON‐SXB doses exceeded AASM CST criteria for the percentage of participants with improvement on the CGI‐I score compared to baseline. The proportion of participants with NT1 who exceeded the CST was 84.6% (55/65) at Week 3 (6 g), 88.9% (48/54) at Week 8 (7.5 g) and 96.0% (48/50) at Week 13 (9 g; Figure 2B). The proportion of participants with NT2 who exceeded the CST was 68.2% (15/22) at Week 3 (6 g), 85.7% (18/21) at Week 8 (7.5 g) and 84.2% (16/19) at Week 13 (9 g). Results for CGI‐I rating with the placebo group are presented in the (Figure S2A,B).

#### 3.2.3 Number of Cataplexy Episodes

**FIGURE 3:**
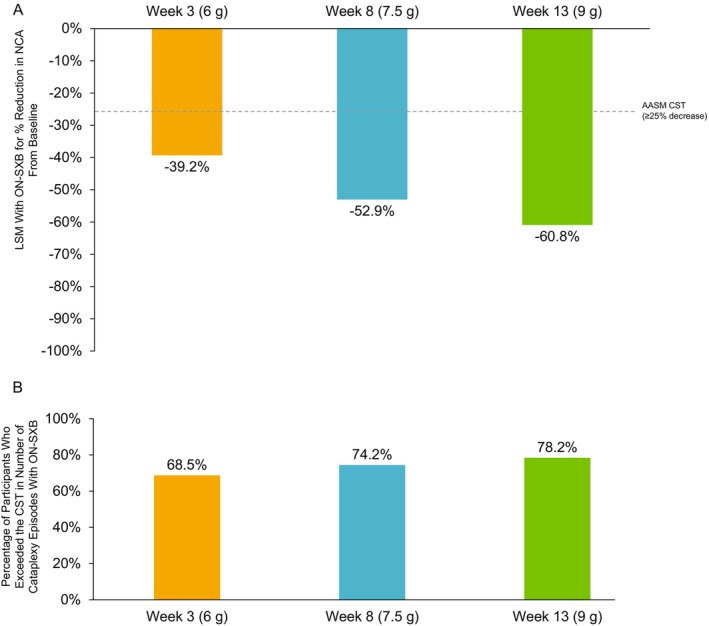
Clinically significant improvements in number of cataplexy episodes with ON‐SXB. LSM change from baseline in percentage reduction in number of cataplexy episodes with ON‐SXB with the associated CST (A) and percentage of participants who exceeded the CST in the mITT population (B). AASM CST is shown as a dotted line. AASM, American Academy of Sleep Medicine; CST, clinical significance threshold; LSM, least squares mean; mITT, modified intent‐to‐treat; NCA, number of cataplexy episodes; ON‐SXB, once‐nightly sodium oxybate.

Among participants with NT1, the LSM percentage reduction in the number of cataplexy episodes from baseline with ON‐SXB at Week 3 (6 g), Week 8 (7.5 g) and Week 13 (9 g) was 39.2%, 52.9%, and 60.8%, respectively, exceeding the AASM CST criteria (Figure 3A). LSM reduction in change from baseline in the number of cataplexy episodes with ON‐SXB versus placebo was 26.0%, 34.2%, and 36.1% at Week 3 (6 g), Week 8 (7.5 g) and Week 13 (9 g), respectively, again meeting AASM CST criteria and demonstrating statistically significant improvements with ON‐SXB versus placebo (*p* < 0.001). The percentage of participants who received ON‐SXB and exceeded the CST was 68.5% (50/73) at Week 3 (6 g), 74.2% (49/66) at Week 8 (7.5 g) and 78.2% (43/55) at Week 13 (9 g) (Figure 3B). Results for the number of cataplexy episodes with the placebo group are presented in the (Figure S3A,B).

#### 3.2.4 ESS

**FIGURE 4:**
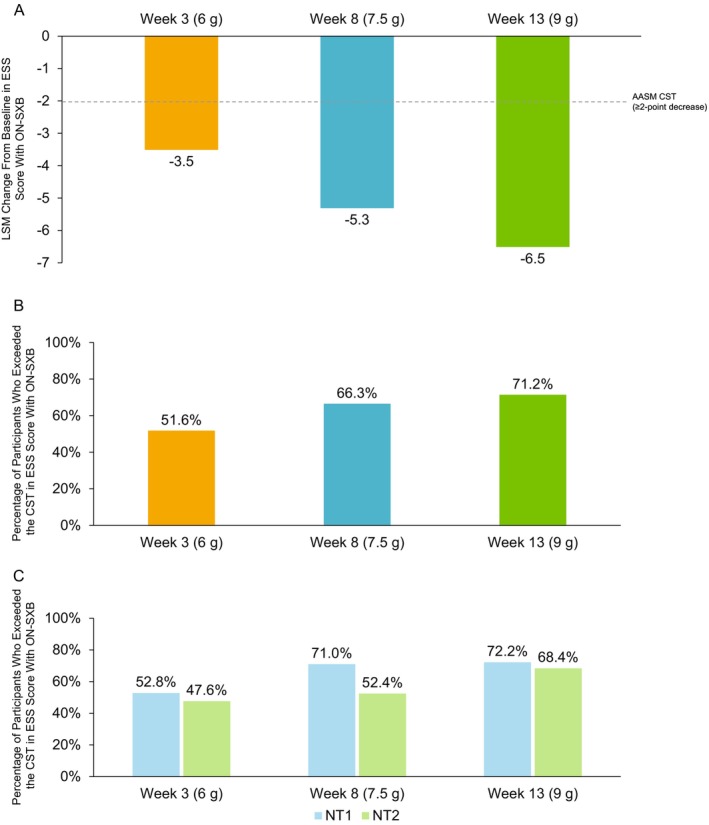
Clinically significant improvements in ESS score with ON‐SXB. LSM change from baseline in ESS score with ON‐SXB with the associated CST (A) and percentage of participants who exceeded the CST in the mITT population (B) and percentage of participants who exceeded the CST in the mITT population by narcolepsy type (C). AASM CST is shown as a dotted line. AASM, American Academy of Sleep Medicine; CST, clinical significance threshold; ESS, Epworth Sleepiness Scale; LSM, least squares mean; mITT, modified intent‐to‐treat; NT1, narcolepsy type 1; NT2, narcolepsy type 2; ON‐SXB, once‐nightly sodium oxybate.

LSM change from baseline in ESS score with ON‐SXB at Week 3 (6 g), Week 8 (7.5 g), and Week 13 (9 g) was −3.5, −5.3 and −6.5, respectively. The decreases exceeded AASM CST criteria for all studied ON‐SXB doses (Figure 4A). Differences in LSM change (95% CI) from baseline in ESS scores were −2.11 (−3.2 to −0.9), −3.2 (−4.7 to −1.6), and −3.9 (−5.5 to −2.3) for ON‐SXB at Week 3 (6 g), Week 8 (7.5 g) and Week 13 (9 g) compared to placebo, respectively. All studied doses of ON‐SXB compared to placebo met AASM CST criteria and were statistically significant in favour of ON‐SXB (*p* ≤ 0.001). The percentage of participants who received ON‐SXB and exceeded the CST was 51.6% (48/93) at Week 3 (6 g), 66.3% (55/83) at Week 8 (7.5 g) and 71.2% (52/73) at Week 13 (9 g) (Figure 4B). The proportion of participants with NT1 who exceeded the CST was 52.8% (38/72) at Week 3 (6 g), 71.0% (44/62) at Week 8 (7.5 g) and 72.2% (39/54) at Week 13 (9 g; Figure 4C). The proportion of participants with NT2 who exceeded the CST was 47.6% (10/21) at Week 3 (6 g), 52.4% (11/21) at Week 8 (7.5 g) and 68.4% (13/19) at Week 13 (9 g). Results for ESS score with the placebo group are presented in the (Figures S4A–C).

#### 3.2.5 Composite of Multiple Disease State Measures

**FIGURE 5:**
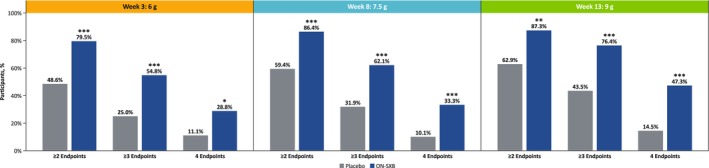
Clinically significant improvements in multiple disease state measures at Week 3, Week 8 and Week 13. ON‐SXB, once‐nightly sodium oxybate. *p ≤ 0.05, **p ≤ 0.01, ***p ≤ 0.001.

At all tested doses, a statistically significantly greater proportion of participants with NT1 who received ON‐SXB versus placebo experienced improvements in ≥ 2, ≥ 3, or 4 clinical endpoints per AASM CST criteria (*p* < 0.05; Figure 5). At Week 3 (6 g), significantly more participants with NT1 who were treated with ON‐SXB versus placebo demonstrated clinical improvements on ≥ 2 (79.5% [58/73] vs. 48.6% [35/72]; *p* < 0.001), ≥ 3 (54.8% [40/73] vs. 25.0% [18/72]; *p* < 0.001), or 4 (28.8% [21/73] vs. 11.1% [8/72]; *p* = 0.012) endpoints. Similarly, at Week 8 (7.5 g), a statistically significantly larger proportion of participants with NT1 who were treated with ON‐SXB versus placebo demonstrated improvements on ≥ 2 (86.4% [57/66] vs. 59.4% [41/69]; *p* < 0.001), ≥ 3 (62.1% [41/66] vs. 31.9% [22/69]; *p* < 0.001), or 4 (33.3% [22/66] vs. 10.1% [7/69]; *p* = 0.001) endpoints. At Week 13 (9 g), this trend continued, as a statistically significantly greater number of participants with NT1 who were treated with ON‐SXB versus placebo showed clinical improvements on ≥ 2 (87.3% [48/55] vs. 62.9% [39/62]; *p* = 0.003), ≥ 3 (76.4% [42/55] vs. 43.5% [27/62]; *p* < 0.001), or all 4 (47.3% [26/55] vs. 14.5% [9/62]; *p* < 0.001) endpoints.

## 4 Discussion

ON‐SXB at doses of 6, 7.5 and 9 g demonstrated clinically significant improvement from baseline in measures of EDS, disease severity, and cataplexy per AASM‐established CST criteria (Kushida et al. 2022; Maski et al. 2021b). Further, more participants who received ON‐SXB experienced clinically significant improvements per AASM CSTs in ≥ 2, ≥ 3, or all 4 clinical endpoints compared with placebo at all tested doses (Maski et al. 2021b). Considering the multitude of symptoms that people with narcolepsy may experience (Drakatos and Leschziner 2014), evaluating a composite of multiple symptom measures may be useful for clinicians in setting expectations of treatment benefit with ON‐SXB.

Although the 2021 AASM CPGs did not include recommendations for ON‐SXB because the data were not yet available, these results align with the strong recommendation by AASM for twice‐nightly SXB (Maski et al. 2021a). The AASM CPGs are reviewed at least every 5 years and updated as necessary (American Academy of Sleep Medicine 2024), and these findings support the inclusion of ON‐SXB in the next AASM CPG update. Similarly, the European Narcolepsy Network, European Academy of Neurology, and European Sleep Research Society guidelines were also last updated in 2021 (Bassetti et al. 2021; Kushida et al. 2022). The European guidelines include a strong recommendation for SXB for DNS (Bassetti et al. 2021; Maski et al. 2021a), as did the 2007 AASM Practice Parameters (Morgenthaler et al. 2007). However, the current AASM CPGs do not describe recommendations for DNS (Maski et al. 2021a). The AASM task force included sleep quality as an outcome by patient population, intervention, comparison, and outcomes question for narcolepsy, and among the CSTs, a decrease of three points on the Pittsburgh Sleep Quality Index (PSQI) was included (Maski et al. 2021b). Although the PSQI was not measured in REST‐ON (Kushida et al. 2022), previously published ON‐SXB data showed significant improvements in both objective (i.e., nocturnal arousals and sleep stage shifts) and subjective (i.e., sleep quality and refreshing nature of sleep) prespecified DNS endpoints from the REST‐ON trial (Roth et al. 2022), aligning with relevant measurements proposed in a recent seminal DNS review (Maski et al. 2022).

Previous post hoc analyses from REST‐ON broadly characterised the effects of ON‐SXB on cataplexy and objective measures of sleepiness and alertness, showing that a significantly greater proportion of participants with NT1 who received ON‐SXB vs. placebo experienced a 25% reduction from baseline in the mean number of cataplexy episodes (Thorpy, Kushida, Bogan, Ajayi, et al. 2024a) and a significantly greater proportion of participants (both NT1 and NT2) exceeded a ≥ 2‐min increase on the MWT, meeting the AASM CSTs for both endpoints (Maski et al. 2021b; Thorpy, Kushida, Bogan, Winkelman, et al. 2024b). The present findings extend this work by focusing on the clinically meaningful effects of ON‐SXB across four different endpoints, including cataplexy and the MWT. These findings highlight the benefits of ON‐SXB in clinical practice. Together, the current and previous post hoc findings demonstrate that the improvements with ON‐SXB treatment are not only statistically significant but also clinically meaningful. In some participants, the observed benefits greatly exceeded the AASM CSTs, suggesting that individuals with narcolepsy treated with ON‐SXB may experience substantial improvement beyond what is typically considered clinically relevant.

CSTs help inform clinical practice decisions (Mishra et al. 2023) and provide an important framework for discussions with clinicians and patients to better understand and recognise improvements in disease outcome measures. Use of thresholds that establish minimal clinically important differences (MCIDs) is increasingly common in clinical trials and research, as these MCIDs incorporate the patient's perspective on treatment effectiveness. MCIDs have been developed for sleep disorder severity scales, including the Narcolepsy Severity Scale for NT1 (Barateau et al. 2021; Dauvilliers et al. 2020, 2017) and NT2 (Barateau et al. 2024), the ESS (Barateau et al. 2024), and the Idiopathic Hypersomnia Severity Scale (Dauvilliers et al. 2019; Rassu et al. 2022). In individuals with NT2, the estimated MCID for ESS is a > 2.1‐point decrease using the Cohen's effect size rule (Barateau et al. 2024). Further research assessing MCIDs on the different scales for NT1 and NT2 is warranted. Notably, the 2021 AASM guidelines were developed using data from trials of twice‐nightly SXB that either enrolled patients with NT1 only or unspecified narcolepsy (Maski et al. 2021b). Previous post hoc analyses from REST‐ON demonstrated consistent efficacy of ON‐SXB in both NT1 and NT2 (Dauvilliers et al. 2023). The data presented herein bolster these previous findings, further demonstrating that ON‐SXB treatment was associated with clinically meaningful improvements in both patients with NT1 and NT2.

Importantly, the robust efficacy of ON‐SXB is demonstrated by clinical improvements across multiple outcomes, including objective and subjective measures of EDS (i.e., mean sleep latency on the MWT and ESS score), clinician rating of improvement (CGI‐I), and number of cataplexy episodes (NT1 only). Differing sensitivities across these assessments likely reflect the complexity of narcolepsy and varied treatment responses among individuals with narcolepsy, particularly given the lack of tools validated specifically in patients with narcolepsy (Kallweit et al. 2017; Schokman et al. 2022). Although the evaluation of ON‐SXB efficacy in the REST‐ON clinical trial was based on differences compared to placebo, in clinical practice, treatment effectiveness is generally compared to an individual's baseline. The AASM guidelines also allow for comparison of efficacy to baseline (Maski et al. 2021b). Applying the CSTs in this manner demonstrates a larger change, as the placebo effect is removed, better reflecting clinical practice. Compared to baseline, CSTs were exceeded for all reported outcomes in which participants received ON‐SXB. Using the stringent placebo‐controlled criteria required for registrational trials, the LSM differences and odds ratios were significantly in favour of ON‐SXB versus placebo for all reported outcomes in REST‐ON (Kushida et al. 2022).

Following the FDA approval of ON‐SXB, there are now 3 different formulations of oxybate medication approved to treat narcolepsy in the US. Two formulations, twice‐nightly SXB and mixed‐salt (calcium, magnesium, potassium and sodium) oxybates, are immediate‐release oxybate formulations (Jazz Pharmaceuticals 2023a). Owing to the short half‐life, individuals must take the first dose at bedtime and a second dose 2.5–4 h later; therefore, people with narcolepsy are routinely required to wake up in the middle of the night to take the second dose of twice‐nightly oxybates. The FDA recognised the third formulation, ON‐SXB, to be a ‘major contribution to patient care’ in the approval decision. As the FDA further described, ‘waking up in the middle of the night to take a second dose is antithetical to the goal of improving sleep’ (US Food 2023). Further, this dosing regimen poses the risk of accidentally taking the second dose < 2.5 h after the first, which has been associated with emergency department visits and hospitalisation (Bogan et al. 2021; Gudeman and Burroughs 2023; Mayer et al. 2018; McIntosh and Mayeux 2024).

Although this post hoc analysis reports outcomes at three different time points, it did not assess the long‐term sustainability of ON‐SXB efficacy at specific doses. Therefore, it is difficult to assess whether the improvements observed at Week 13 were attributable to the dose escalation from 6 g to 7.5 g to 9 g or to the extended duration of ON‐SXB treatment. This analysis did not account for potential differences based on the use of alerting agents; however, a previous post hoc analysis of REST‐ON demonstrated the consistent efficacy of ON‐SXB on daytime narcolepsy symptoms regardless of concomitant alerting agent use (Dauvilliers et al. 2024).

## 5 Conclusion

ON‐SXB at doses of 6, 7.5 and 9 g versus placebo exceeded AASM‐established CSTs and demonstrated statistically significant improvements (*p* < 0.001) in measures of MWT, ESS, CGI‐I and number of weekly cataplexy episodes. Further, a statistically significant number of participants who received ON‐SXB compared with placebo experienced clinically significant improvements across multiple narcolepsy outcome measures. Results from this post hoc analysis extend the primary results from REST‐ON and suggest that ON‐SXB treatment leads to meaningful improvements in narcolepsy symptoms in clinical practice. These findings may help guide patient expectations for treatment benefit with ON‐SXB.

## Author Contributions

**Luis E. Ortiz:** writing – review and editing. **Anne Marie Morse:** writing – review and editing. **Michael J. Thorpy:** writing – review and editing. **Clete A. Kushida:** writing – review and editing. **John Harsh:** writing – review and editing. **Thomas Roth:** writing – review and editing. **Jennifer Gudeman:** writing – review and editing. **Yves Dauvilliers:** writing – review and editing.

## Ethics Statement

The trial was conducted in compliance with the ethical principles of the Declaration of Helsinki, Good Clinical Practice guidelines, International Council for Harmonisation guidelines, applicable national and local laws and regulatory requirements. Institutional review boards approved the protocol of the REST‐ON trial.

## Consent

All adult participants (aged ≥ 18 years) provided written informed consent. If participants were 16 or 17 years of age, both the participant and their legally authorised representative provided informed consent.

## Conflicts of Interest

The funding sponsor of this study had a role in the choice of research project; design of the study; in the collection, analyses, and/or interpretation of data; in the writing of the manuscript; and in the decision to publish the results. L.E.O. is a consultant to Harmony Biosciences and has served on advisory boards for Avadel Pharmaceuticals, Harmony Biosciences and Jazz Pharmaceuticals. A.M.M. has served as a consultant, speaker, and/or on advisory boards for Avadel Pharmaceuticals, Eisai, Harmony Biosciences, Jazz Pharmaceuticals, Alkermes and Takeda Pharmaceutical Co; has received grant funding from National Institutes of Health, UCB Pharmaceuticals, Jazz Pharmaceuticals, ResMed Foundation, Coverys Foundation, Harmony Biosciences and Geisinger Health Plan; is the CEO of DAMM Good Sleep LLC; and serves as an advisor for Neura Health and Floraworks. M.J.T. has served as a consultant or on advisory boards for Axsome Therapeutics, Balance Therapeutics, Eisai, Avadel Pharmaceuticals, Harmony Biosciences, Jazz Pharmaceuticals, NLS Pharmaceuticals, Suven Life Sciences Ltd., and Takeda Pharmaceutical Co. C.A.K. is a consultant for Avadel Pharmaceuticals and XW Pharma. J.H. declares no conflicts of interest. T.R. is a consultant for Jazz Pharmaceuticals, Takeda Pharmaceutical Co., Orexo, Avadel Pharmaceuticals, Eisai, Merck & Co., and Idorsia. J.G. is an employee of Avadel Pharmaceuticals. Y.D. has served as a consultant or on advisory boards for Avadel Pharmaceuticals, Bioprojet, Centessa, Harmony Biosciences, Idorsia, Jazz Pharmaceuticals and Takeda Pharmaceutical Co.

## Supporting information


**Data S1:** Supporting Information.

## Data Availability

The data underlying this article will be shared on reasonable request to the corresponding author.
