# Supplementary material for: Once‐Nightly Sodium Oxybate Meets American Academy of Sleep Medicine Criteria for Treatment of Narcolepsy
Source: J Sleep Res. 2025 Aug 25;35(2):e70189. doi: 10.1111/jsr.70189 (PMC13003262; doi:10.1111/jsr.70189)

# Supplementary Appendix

Once-Nightly Sodium Oxybate Meets the American Academy of Sleep Medicine Criteria for Treatment of Narcolepsy

**Authors**: Luis E. Ortiz, MD^1^; Anne Marie Morse, DO^2^; Michael J. Thorpy, MD^3^; Clete A. Kushida, MD, PhD^4^; John Harsh, PhD^5^; Thomas Roth, PhD^6^; Jennifer Gudeman, PharmD^7^; Yves Dauvilliers, MD, PhD^8^

**Affiliations**: ^1^Johns Hopkins Medical Institutions, Johns Hopkins All Children’s Hospital, St. Petersburg, FL, USA; ^2^Geisinger Commonwealth School of Medicine, Geisinger Medical Center, Janet Weis Children’s Hospital, Danville, PA, USA; ^3^Albert Einstein College of Medicine, Montefiore Medical Center, New York, NY, USA; ^4^Stanford University School of Medicine, Stanford, CA, USA; ^5^Colorado Sleep Institute, Boulder, CO, USA; ^6^Sleep Disorders and Research Center, Henry Ford Health System, Detroit, MI, USA; ^7^Avadel Pharmaceuticals, Chesterfield, MO, USA; ^8^Sleep-Wake Disorders Center, Department of Neurology, Gui-de-Chauliac Hospital, Institute for Neurosciences of Montpellier INM, INSERM, University of Montpellier, Montpellier, France

**Supplementary Figure 1.** **Changes in Mean Sleep Latency on the MWT With ON-SXB and Placebo.** LSM change from baseline with ON-SXB and placebo in mean sleep latency on MWT with the associated CST (A), percentage of participants who exceeded the CST in the mITT population (B), and percentage of participants who exceeded the CST in the mITT population by narcolepsy type (C). CST, clinical significance threshold; LSM, least squares mean; mITT, modified intent-to-treat; MWT, Maintenance of Wakefulness Test; NT1, narcolepsy type 1; NT2, narcolepsy type 2**;** ON-SXB, once-nightly sodium oxybate.

**A.**


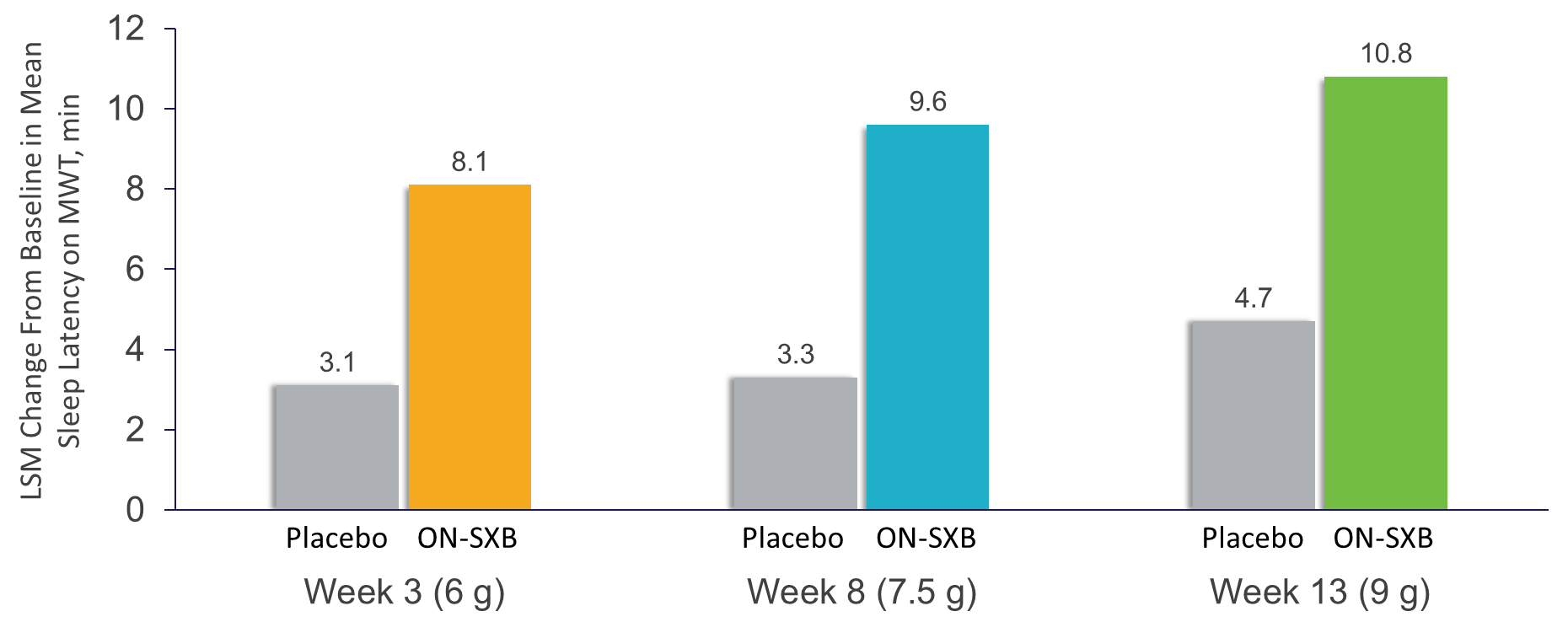


**B.**


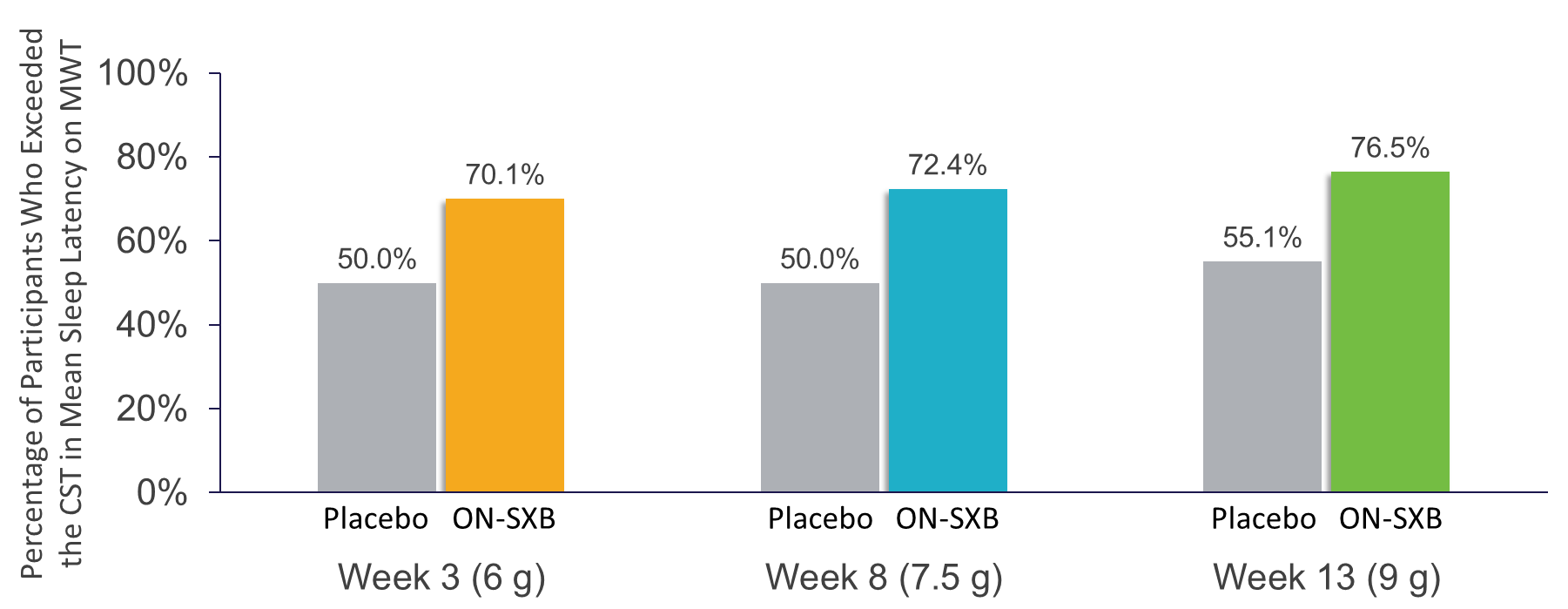


**C.
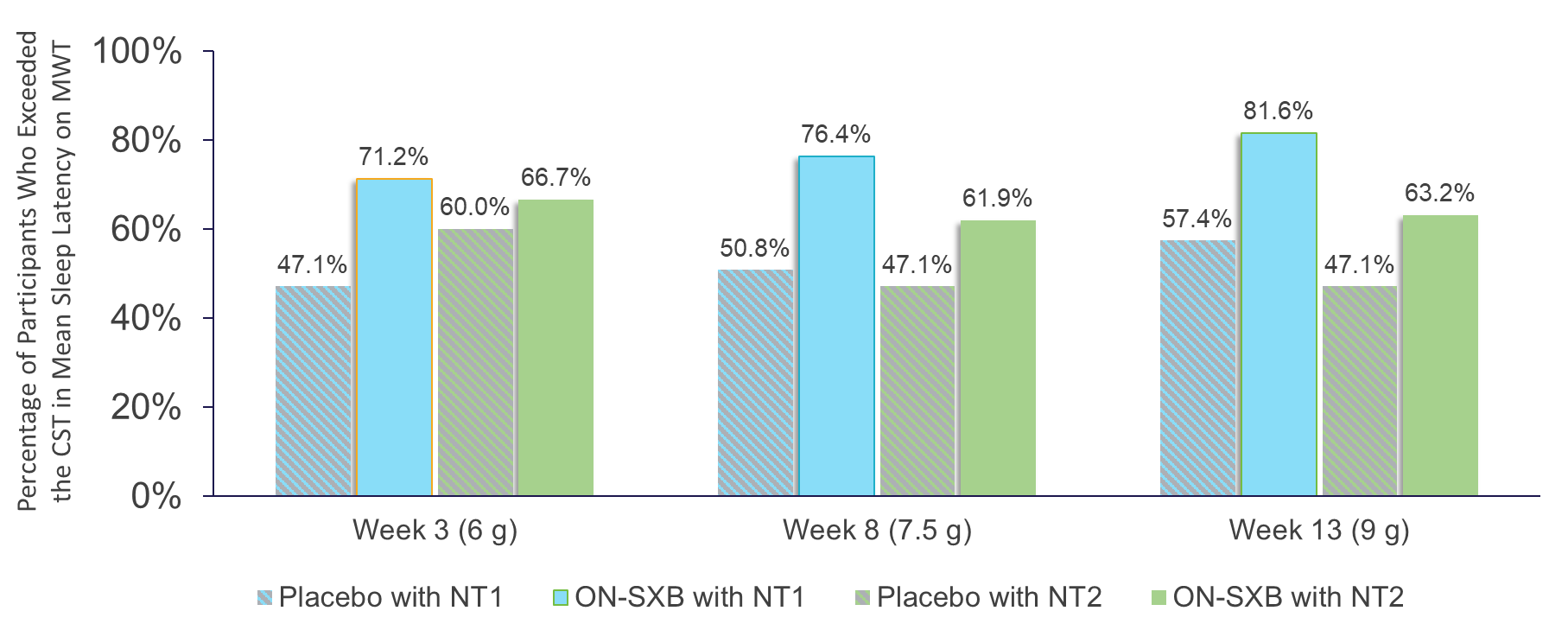
**

Supplementary Figure 2. Changes in CGI-I Rating With ON-SXB and Placebo. Percentage of participants with very much, much, or minimal improvement on CGI-I with ON-SXB and placebo and the associated CST in the mITT population (A) and percentage of participants who exceeded the CST in the mITT population by narcolepsy type (B). CGI-I, Clinical Global Impression of Improvement; CST, clinical significance threshold; mITT, modified intent-to-treat; NT1, narcolepsy type 1; NT2, narcolepsy type 2; ON-SXB, once-nightly sodium oxybate.

**A.**


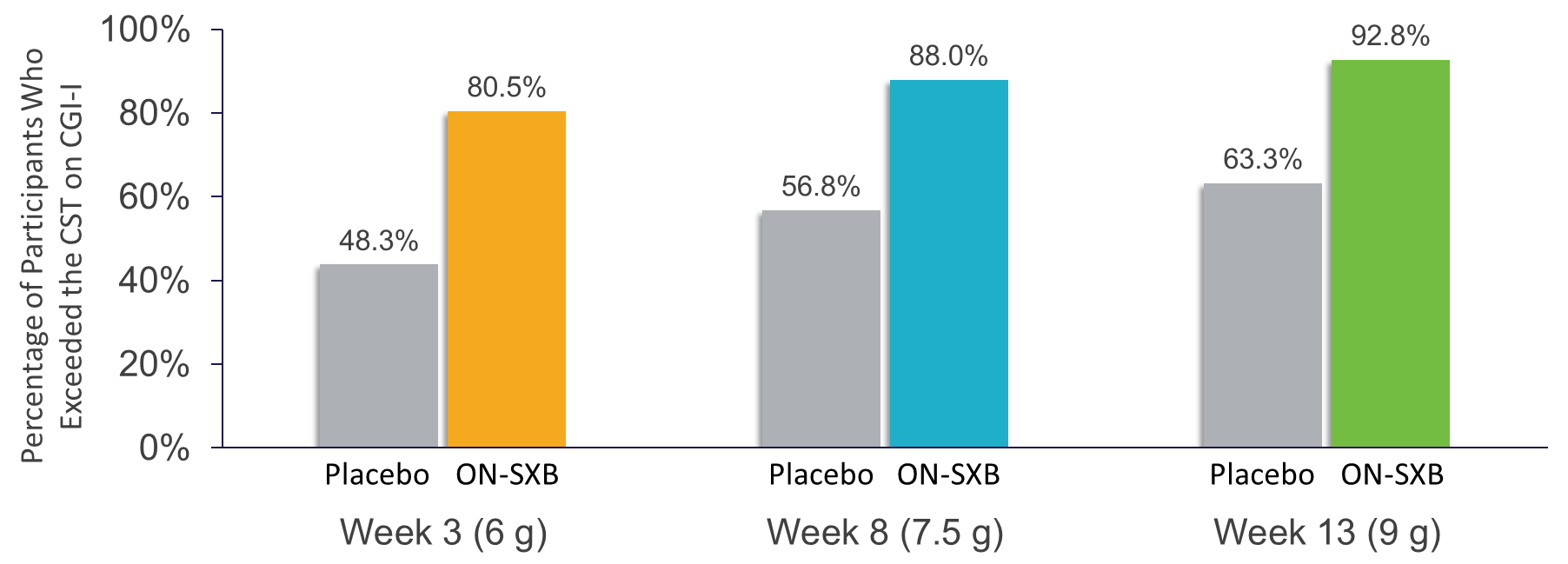


**B.
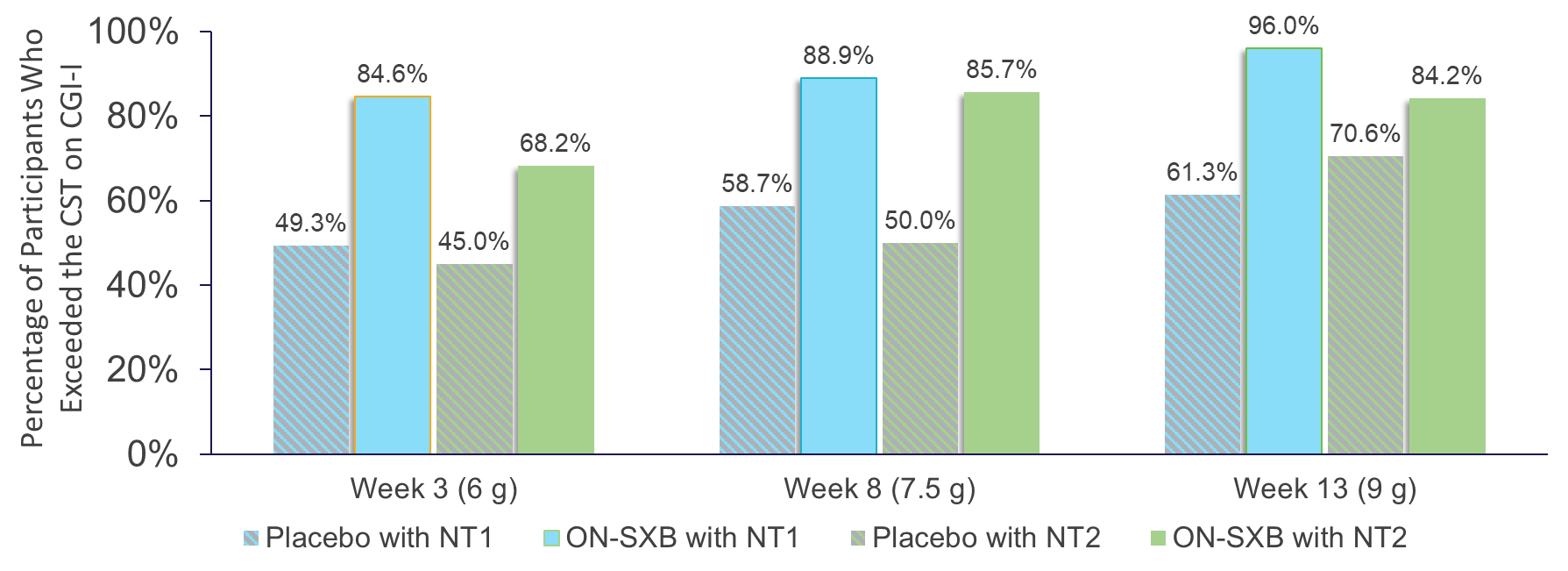
**

Supplementary Figure 3. Changes in Number of Cataplexy Episodes With ON-SXB and Placebo. LSM change from baseline in percentage reduction in number of cataplexy episodes with ON-SXB and placebo with the associated CST (A) and percentage of participants who exceeded the CST in the mITT population (B). CST, clinical significance threshold; LSM, least squares mean; mITT, modified intent-to-treat; NCA, number of cataplexy episodes; ON-SXB, once-nightly sodium oxybate.

**A.**


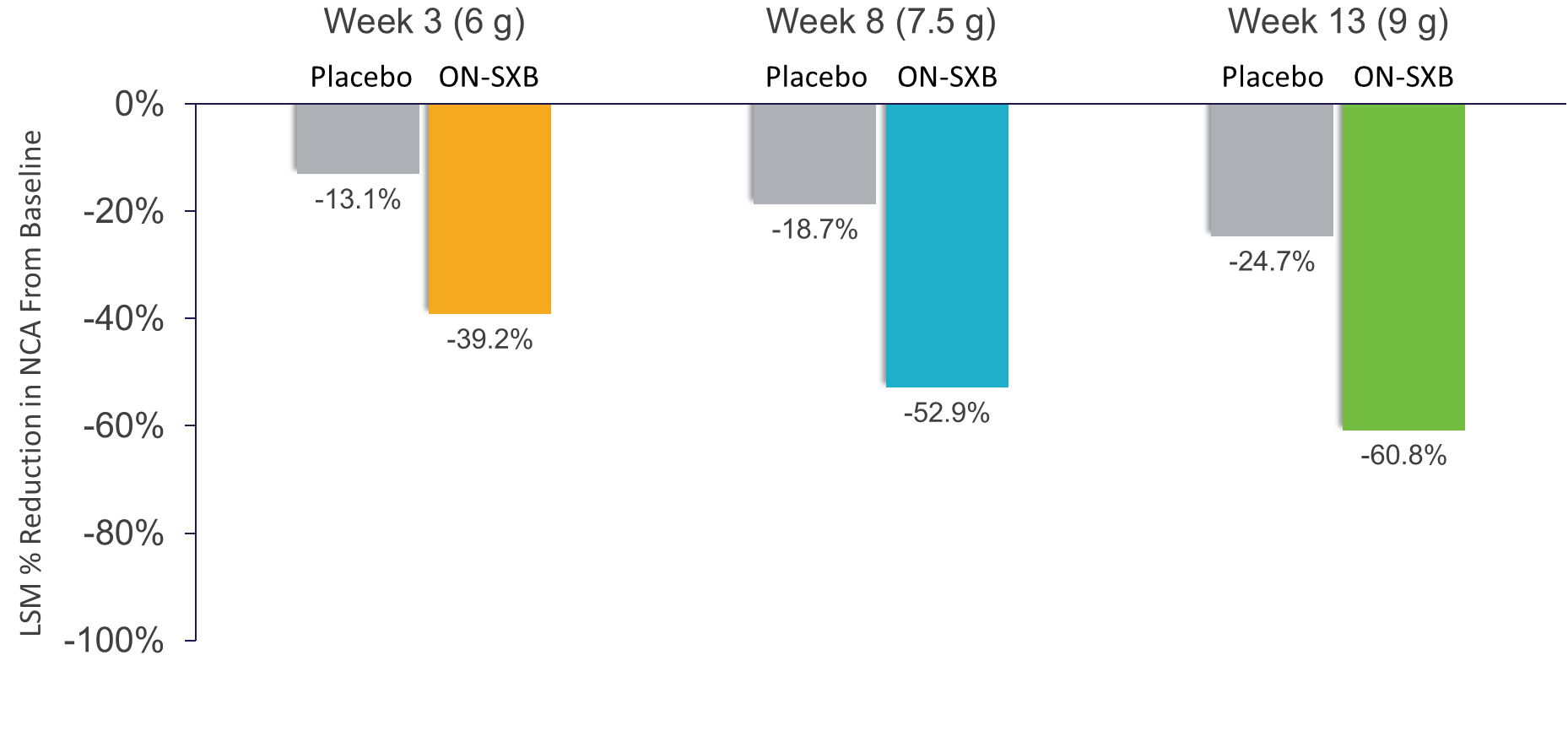


**B.**


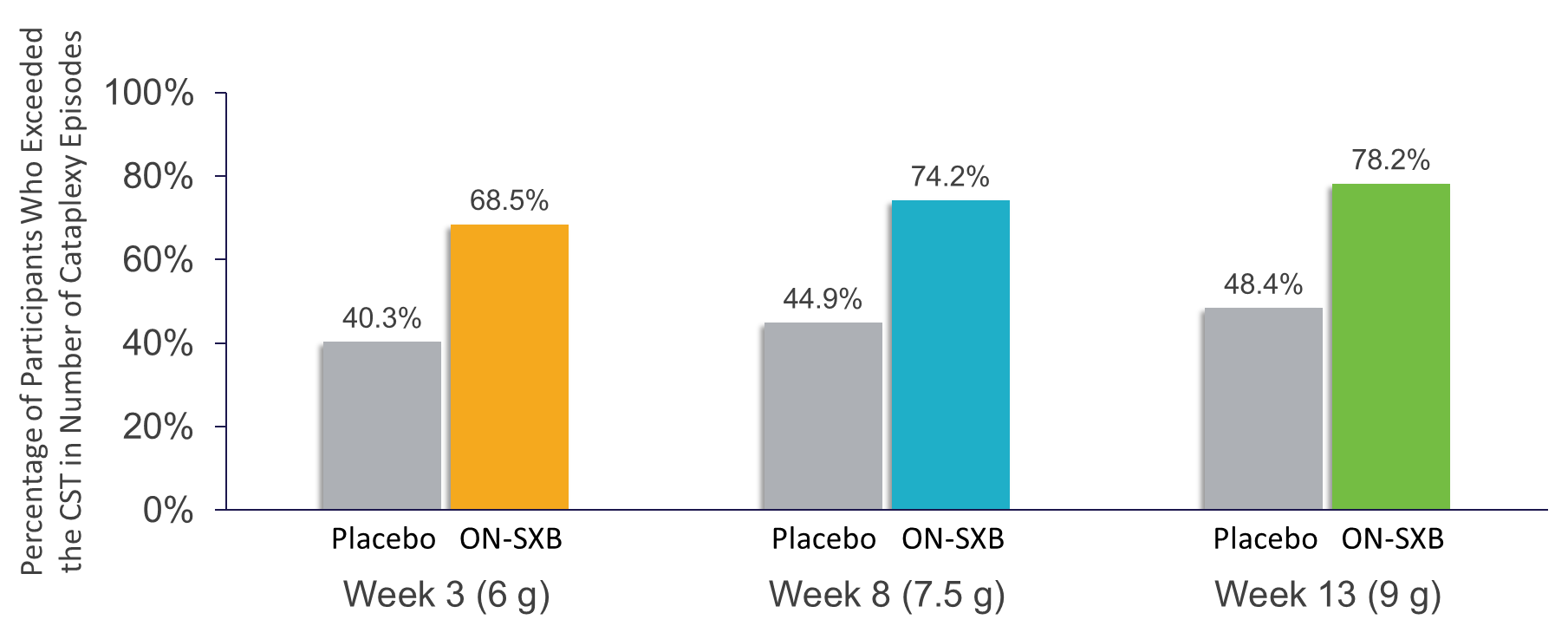


Supplementary Figure 4. Changes in ESS Score With ON-SXB and Placebo. LSM change from baseline in ESS score with ON-SXB and placebo with the associated CST (A) and percentage of participants who exceeded the CST in the mITT population (B) and percentage of participants who exceeded the CST in the mITT population by narcolepsy type (C). CST, clinical significance threshold; ESS, Epworth Sleepiness Scale; LSM, least squares mean; mITT, modified intent-to-treat; NT1, narcolepsy type 1; NT2, narcolepsy type 2; ON-SXB, once-nightly sodium oxybate.

**A.**


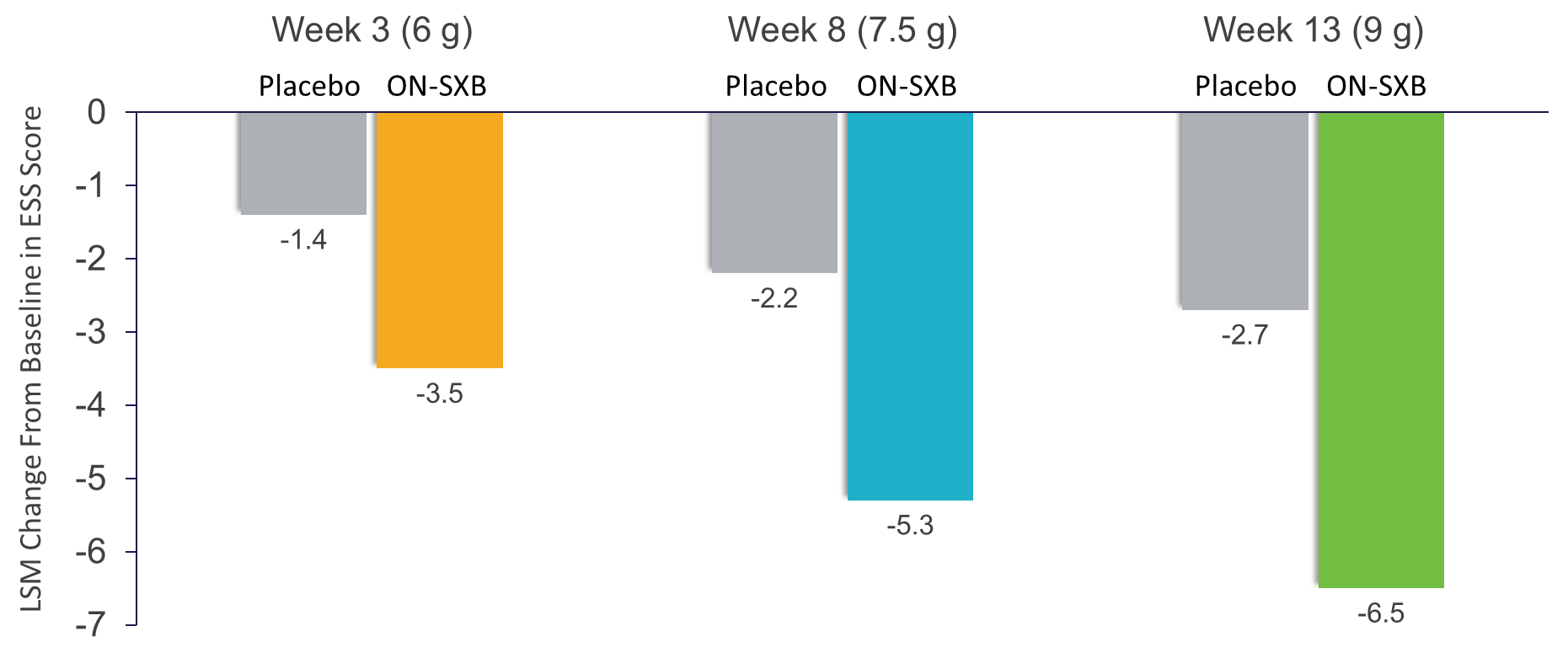


**B.
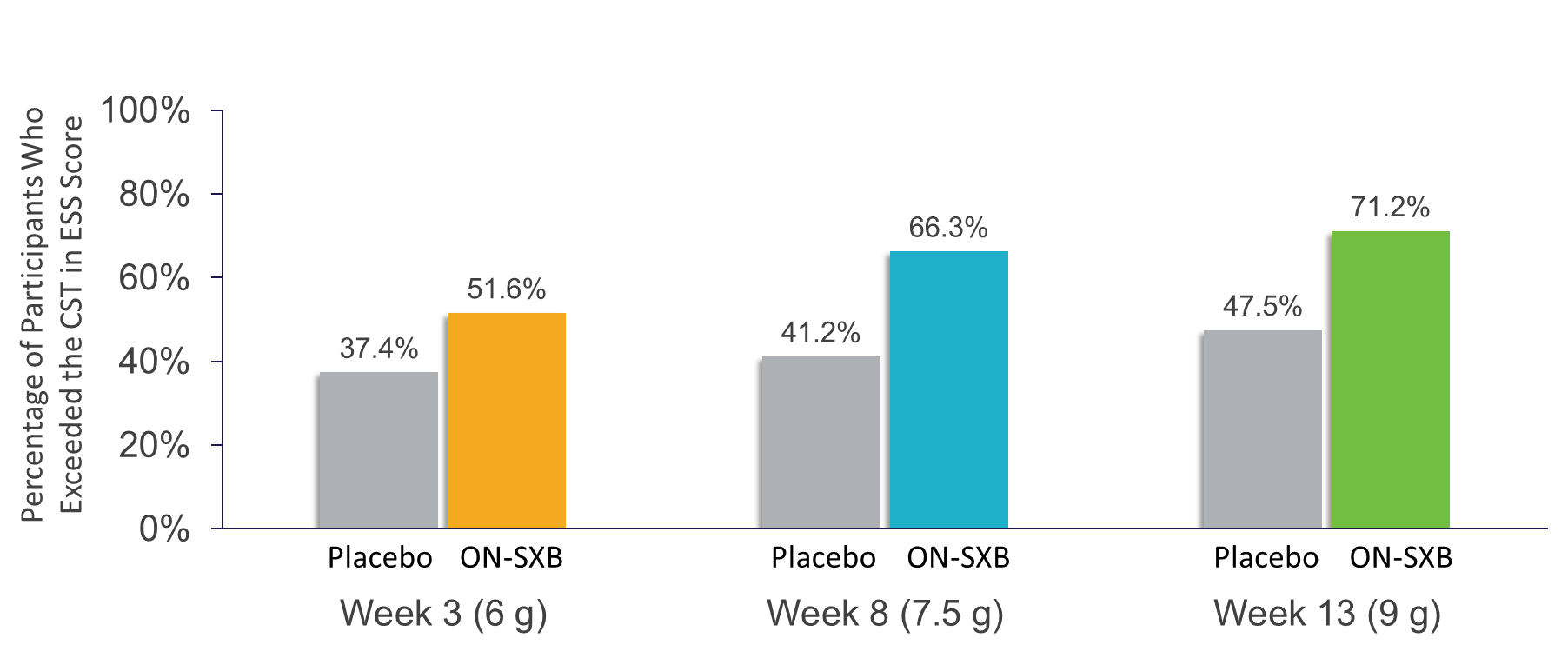
**

**C.**


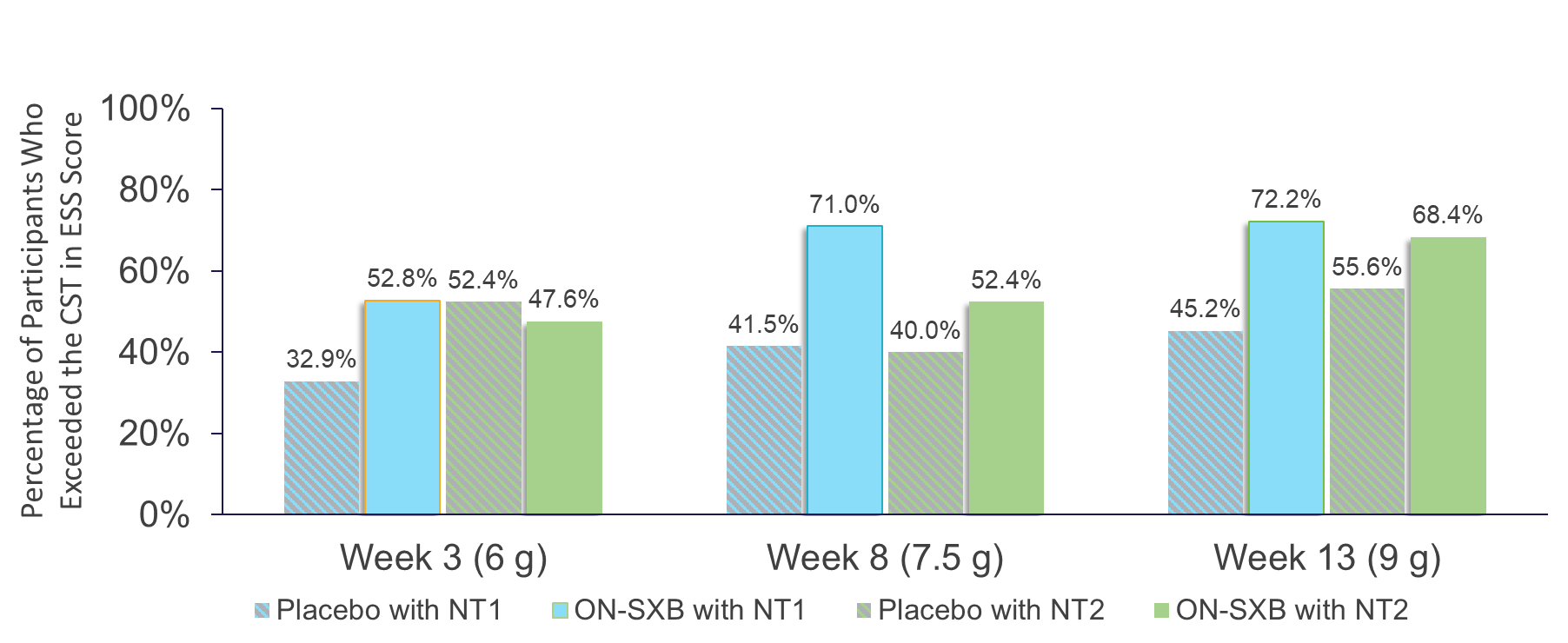

Supplement: Supplementary file 1 — Data S1: Supporting Information. [file JSR-35-e70189-s001.docx]
